# Beyond Extracellular Vesicles: Hybrid Membrane Nanovesicles as Emerging Advanced Tools for Biomedical Applications

**DOI:** 10.1002/advs.202303617

**Published:** 2023-09-25

**Authors:** Meng Sun, Jiani Yang, Yueyun Fan, Yinfeng Zhang, Jian Sun, Min Hu, Ke Sun, Jinfeng Zhang

**Affiliations:** ^1^ Key Laboratory of Molecular Medicine and Biotherapy School of Life Sciences Beijing Institute of Technology Beijing 100081 P. R. China; ^2^ International Medical Center Beijing Friendship Hospital Capital Medical University Beijing 100050 P. R. China; ^3^ Department of Hepatobiliary Surgery Jinan University First Affiliated Hospital Guangzhou 510630 P. R. China; ^4^ Department of Urinary surgery The First Affiliated Hospital of Zhengzhou University Zhengzhou Henan 450052 China

**Keywords:** diagnosis and treatment, exosomes, extracellular vesicles, hybrid membrane nanovesicles, membrane fusion

## Abstract

Extracellular vesicles (EVs), involved in essential physiological and pathological processes of the organism, have emerged as powerful tools for disease treatment owing to their unique natural biological characteristics and artificially acquired advantages. However, the limited targeting ability, insufficient production yield, and low drug‐loading capability of natural simplex EVs have greatly hindered their development in clinical translation. Therefore, the establishment of multifunctional hybrid membrane nanovesicles (HMNVs) with favorable adaptability and flexibility has become the key to expanding the practical application of EVs. This timely review summarizes the current progress of HMNVs for biomedical applications. Different HMNVs preparation strategies including physical, chemical, and chimera approaches are first discussed. This review then individually describes the diverse types of HMNVs based on homologous or heterologous cell membrane substances, a fusion of cell membrane and liposome, as well as a fusion of cell membrane and bacterial membrane. Subsequently, a specific emphasis is placed on the highlight of biological applications of the HMNVs toward various diseases with representative examples. Finally, ongoing challenges and prospects of the currently developed HMNVs in clinical translational applications are briefly presented. This review will not only stimulate broad interest among researchers from diverse disciplines but also provide valuable insights for the development of promising nanoplatforms in precision medicine.

## Graphical abstract

This review explores the diverse strategies for fabricating hybrid membrane nanovesicles (HMNVs) and the various types of HMNVs based on membrane components and chimera biotechnology. It also summarizes the current advancements in biomedical applications of HMNVs, including diagnosis, bioimaging, and treatment of different diseases. Finally, it presents the ongoing challenges and prospects for clinical translation of currently developed HMNVs.

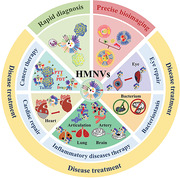

## 1 Introduction

With the speedy advancement of emerging biotechnologies, extracellular vesicles (EVs) with low immunogenicity, superior load capability, facile modifiability, and multiple natural bioproperties, play an increasingly critical role in biomedical applications such as preclinical diagnosis, diseases treatment, and prognosis assessment.^[^ ^1^ ^]^ EVs, mainly composed of lipid bilayers, are actively secreted by almost all types of cells and thus widely present in various bodily fluids, such as blood, tears, urine, saliva, breast milk, and ascites. There are a variety of bioactive components inside EVs, including nucleic acids (e.g., DNA, mRNA, microRNA, lncRNA, etc.), proteins, metabolic enzymes, lipids, and other active macromolecules. More attractively, numerous surface biomarkers are rich in EVs membrane, including tetraspanins (e.g., CD9, CD63, CD81), adhesion molecules (e.g., integrins, selectins), Chaperones (e.g., HSP70, HSP90), as well as other membrane transport and fusion proteins. Such abundant bioactive components exited within or on the surface of EVs not only allow the EVs to maintain intrinsic biological properties inherited from their parental cells, enabling them to participate in cellular information transfer and material exchange,^[^ ^2^ ^]^ but also endow EVs with further editable and targetable capabilities.^[^ ^3^ ^]^

At the present stage, EVs are briefly classified into three main categories according to their diverse biogenesis, secretion pathways, and size, namely, exosomes (40–200 nm), microvesicles (200–2000 nm), and apoptotic bodies (500–2000 nm).^[^ ^4^ ^]^ Up to now, all these three types of EVs have been extensively exploited for biological and biomedical applications.^[^ ^1^, ^5^ ^]^ Especially, among them, exosomes have garnered great attention as one of the most promising diagnostic tools, therapeutic agents, and drug delivery vehicles,^[^ ^6^ ^]^ some of which are even for clinical trials. For example, exosomes from autologous‐derived dendritic cells (DCs) loaded with MHC class I peptides were injected into melanoma patients for reducing skin tumors and lymph node lesions, and this phase I trial found symptomatic relief and mild inflammation in these patients. In another case, mesenchymal stem cell (MSC)‐derived exosomes (MSC‐Exos) exhibited significant treatment outcomes in chronic kidney disease during a phase II/phase III clinical pilot study.^[^ ^7^ ^]^ However, despite their indisputable potential, there remain substantial urgent issues about natural EVs that need to be addressed. For example, most naturally secreted EVs lack targeting ability to the intended tissues or organs, resulting in an unsatisfactory distribution profile. Meanwhile, complex isolation methods of EVs lead to poor purification efficiency and low production yield, which significantly hinder their practicability and universality. Besides, low drug loading capability and compromised therapeutic efficacy of unmodified EVs for biomedical application are also their tricky downsides.^[^ ^8^ ^]^

**Figure 1:**
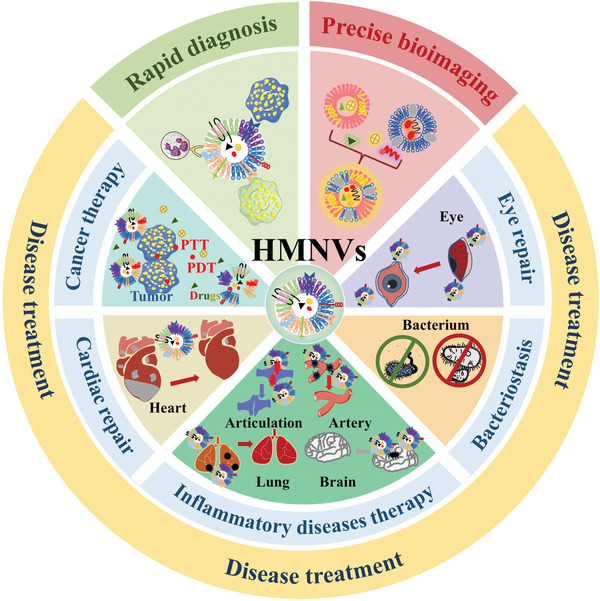
Schematic illustration of the HMNVs for various biomedical applications.

**Figure 2:**
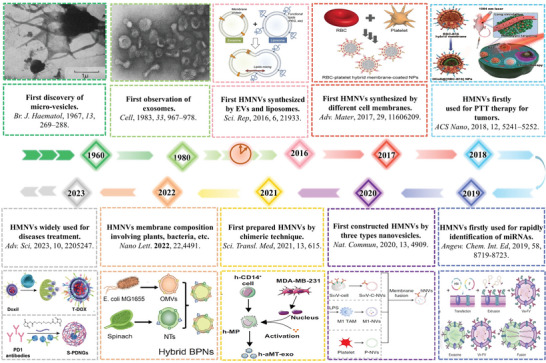
Timeline of representative milestones from EVs to HMNVs fields. Reproduced with permission.^[^ ^10^ ^]^ Copyright 1961, Wiley‐VCH. Reproduced with permission.^[^ ^11^ ^]^ Copyright 1983, Elsevier. Reproduced with permission.^[^ ^12^ ^]^ Copyright 2016, Springer Nature. Reproduced with permission.^[^ ^13^ ^]^ Copyright 2017, Wiley‐VCH. Reproduced with permission.^[^ ^14^ ^]^ Copyright 2018, American Chemical Society. Reproduced with permission.^[^ ^15^ ^]^ Copyright 2019, Wiley‐VCH. Reproduced with permission.^[^ ^16^ ^]^ Copyright 2020, Springer Nature. Reproduced with permission.^[^ ^17^ ^]^ Copyright 2021, AAAS. Reproduced with permission.^[^ ^18^ ^]^ Copyright 2022, American Chemical Society. Reproduced with permission.^[^ ^19^ ^]^ Copyright 2023, Wiley‐VCH.

Fortunately, hybrid membrane nanovesicles (HMNVs), formed by the fusion of homologous and heterologous membrane substances derived from either natural secretion or artificial synthesis, have recently sparked growing interest as promising alternatives to EVs since they can circumvent some inherent problems of EVs.^[^ ^9^ ^]^ For instance, HMNVs are artificially assembled nanovesicles, which can be obtained at a large‐scale grade by extrusion through membrane filters or ultrasound over easily accessible instruments. Moreover, multiple functions and diverse applications can be synchronously performed in one type of HMNVs, not to mention the improved targeting ability. In particular, the fused HMNVs derived from two different types of cells exhibit biological features and overlaid functions from both source cells. More intriguingly, through genetic editing or chemical modification of the biomolecules within HMNVs or cell membrane components on the surface of HMNVs, these nanovesicles can be served as an individually customized platform with well‐defined substances, offering a high level of safety as well as a high degree of variability and more possibilities. Given the above merits, various biomedical applications of the HMNVs are shown in **Figure** **1**. **Figure** **2** illustrates the timeline of representative milestones from EVs to HMNVs fields. In light of the recent progress in the nascent field, this review aimed to provide the latest updates on the development of HMNVs for different biomedical applications, which will appeal to a wide range of scientists and researchers from interdisciplinary fields of chemistry, bioengineering, materials science, nanomedicine, pharmacology, and clinical medicine. More importantly, this review provides insightful perspectives and clear future prospects for the rapid advancement of both engineered EVs and hybrid nanovesicles, which will be of high value to exploit the newly powerful nanoplatforms for personalized and precision medicine.

## 2 Different Strategies for the Preparation of HMNVs

**Figure 3:**
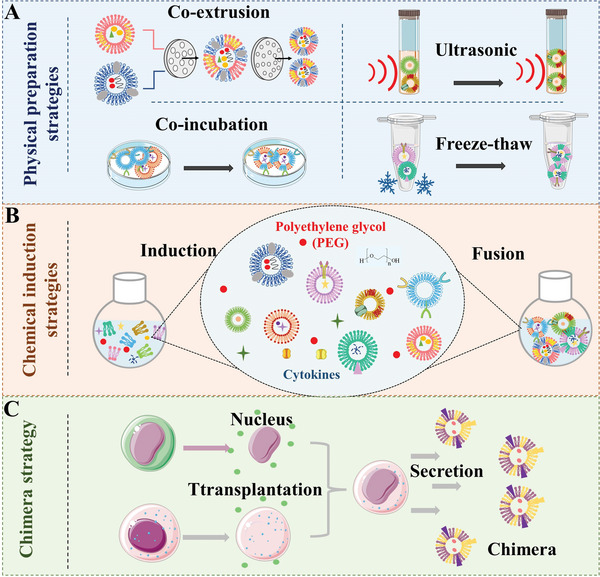
Schematic illustration of three main strategies for HMNVs fabrication, including A) physical preparation strategies, B) chemical induction strategies, and C) chimera strategy.

Doubtlessly, it is crucial to develop reliable HMNVs preparation methods with desirable standardization, reproducibility, scalability, and productivity for promoting the translational utility of the HMNVs in biomedicine. Different from the EVs that are naturally secreted by various source cells, the bioinspired HMNVs are constructed by multiple manual intervention approaches, including but not limited to a membrane fusion of the particular engineered EVs and another homogeneous/heterogeneous cell‐derived EVs, a fusion of cell membrane and liposome, gene transfection method, as well as chimera biotechnology. In general, we reviewed the present preparation strategies for HMNVs and summarized them into three main categories, including physical preparation strategies, chemical induction strategies, and biological transformation strategies (**Figure** **3**). Distinctive advantages and limitations of the current major preparation technologies of HMNVs are summarized in **Table** **1**.

**Table 1:** Advantages and limitations of the current major preparation technologies of the HMNVs.

Approach		Advantages	Limitations	Refs.
Physical preparation strategies	Coextrusion	Excellent fusion efficiency, high loading efficiency, suitable fusion size, satisfactory production yield	Complex processing, rearrangement of membrane surface, membrane damage	[21, 22]
	Freeze–thaw	Simple operation, short operation time, low equipment cost	Aggregation and damage, leakage of contents, difficult separation and purification	[12]
	Sonication	Simple operation, high fusion efficiency, low cost	Structural damage, low drug loading capacity, uncontrolled fusion size	[25]
	Coincubation	Simple operation, no special equipment requirements, harmless to membranes, low cost	Aggregation and damage, low fusion efficiency, time‐consuming, uncontrolled fusion size	[26]
Chemical induction strategies	PEG induction method	Good fusion efficiency, simple operation, low cost	Aggregation, potential chemical damage or contamination, uncontrolled fusion size	[30, 31, 32]
Chimera strategy		Excellent fusion efficiency, good drug loading capacity, precise but safe way, scaled operation	Complex process, expensive equipment cost, in the initial phase, ethical issues	[17]

### 2.1 Physical Preparation Strategies

Coextrusion is the most widespread way in the preparation of HMNVs by using professional instruments like microsqueezer assisted with external forces such as extrusion, push, and pull. For example, uniform‐sized hybrid nanovesicles can be formed when membranes deriving from two different cell sources are passively passed through the filter membrane.^[^ ^20^ ^]^ Li et al. prepared a fusion vesicle of platelet membrane (PMV) and MSC exosomes (MSC‐Exos) by coextrusion for the treatment of myocardial ischemia injury.^[^ ^21^ ^]^ The PMV was extracted by the freeze–thaw method while the MSC‐Exos were extracted by differential centrifugation. The resultant PMV and MSC‐Exos were mixed in the same proportion and were passed through 400 and 200 nm polycarbonate porous membranes in turn. Finally, a fusion vesicle with a size of 138 nm was obtained. The as‐fabricated hybrid EVs not only retained the natural ability of platelets to target the heart injury but also inherited the function of MSC to repair the vascular injury and promote angiogenesis. To gain a high production yield and enhance the tumor‐targeting capability of the HMNVs, Rayamajhi et al. reported a hybrid exosome with a size of ≈177 nm for drug delivery application.^[^ ^22^ ^]^ The hybrid nanovesicles were built by coextrusion, which is composed of macrophage J774A.1‐Exos and artificially synthesized liposomes. In addition, the filtration method has the same principle as the coextrusion for the generation of HMNVs with target size, while the preparation efficiency of filtration can be improved by utilizing an automatic device.^[^ ^23^ ^]^

Freeze–thaw methods, in addition to being commonly used for the acquisition of cell membranes, are also used in the formation of fused EV mimetic hybrid nanovesicles.^[^ ^24^ ^]^ The fusion vesicles are acquired during the temperature cycling of mixed membranous substances. For example, Sato et al. used a more straightforward freeze–thaw strategy to realize fusion.^[^ ^12^ ^]^ In this work, equal proportions of RAW264.7 cell‐derived EVs were mixed with synthetic liposomes with polyethylene glycol (PEG) modification, then the mixture was frozen in liquid nitrogen for 15 min and thawed at room temperature for another 15 min. After repeating the cycle several times, the mixture was turned into a fusion. It was found that the fusions formed by this method are commonly not uniform in size or diameter, so such physical preparation method of targeted HMNVs products can be improved by further cooperating with incubation or purification extrusion. Similar to the freeze–thaw method, the sonication method is also based on the principle of break‐reconstruction, which is easy to operate and often served as an auxiliary manner. As Han et al. reported, after 30 rounds of repeated ultrasound, the therapeutic HMNVs could be obtained from the B16F10 cell membranes.^[^ ^25^ ^]^

Coincubation is a strategy that is least harmful to membrane‐type substances, in which different membrane components are usually placed together and coincubated for a given time at 37 °C. Lin et al. mixed the HEK293FT cell‐derived exosomes with liposomes carrying gene‐editing CRISPR/Cas9 plasmids for coincubation at 37 °C for 12 h.^[^ ^26^ ^]^ In the end, the fusions which could efficiently express the CRISPR/Cas9 system were successfully prepared. Nevertheless, it has taken a long time to prepare HMNVs only by coincubation of mixed membrane substances. As such, coincubation is often used as an auxiliary way to construct a more convenient and labor‐saving preparation strategy for HMNVs.

Apart from the above physical preparation methods to HMNVs, microfluidics,^[^ ^27^ ^]^ nitrogen cavitation,^[^ ^28^ ^]^ and cell blebbing (vesiculation)^[^ ^29^ ^]^ have been gradually utilized in the production and preparation of nanovesicles by virtue of the merits of automation, high throughput, and rapid analysis. Unfortunately, these three techniques have not been used in membrane‐derived HMNVs, which provide promising strategies for efficient preparation of HMNVs in near future.

### 2.2 Chemical Induction Strategies

Compared to the physical preparation methods, chemical induction methods are more common and efficient for HMNVs generation with the merits of easy operation and high fusion efficiency. In particular, PEG is the most widely used chemical agent for inducing the fusion of different types of cell membrane sources. For example, Piffoux et al. constructed a bioresponsive fusion nanovesicles for drug delivery.^[^ ^30^ ^]^ The human umbilical vein endothelial cells derived EVs and functionalized liposomes were triggered by PEG8000 to form HMNVs that retained the original biological properties while improving the drug delivery efficiency. Because different relative molecular weights (8000, 6000, and 3000 g mol^−1^) have diverse catalytic efficiencies, the group explored and found that the induction effect of PEG8000 was the best under these conditions. It provides a theoretical basis for the future use of the PEG induction method.

Furthermore, the PEG induction method has usually been combined with other preparation strategies, such as extrusion, to construct HMNVs with higher fusion rates and more desirable dimensions. Ma et al. first used PEG8000 to modify artificial liposomes loaded with photothermal agents, then they extruded the resultant liposomes with platelet exosomes through a 100 nm filter membrane to obtain the fusion nanovesicles to fully play tumor photothermal therapy.^[^ ^31^ ^]^ Hu et al. prepared fusion membrane vesicles of MSC‐Exos and platelet cell membranes.^[^ ^32^ ^]^ In the fusion process, the mixture was first induced in the presence of PEG (5%) and then extruded through a 200 nm filter to obtain HMNVs with the ability to target the myocardial injury sites.

### 2.3 Chimera Strategy

In addition to the strategies mentioned above, chimera technology, a promising approach, is being applied to the preparation of multifunctional HMNVs. Chimera technology broadly refers to biotechnology that can synthesize a single organism (e.g., tissue, organ, or a person) containing a mixture of cells with disparate genetic backgrounds.^[^ ^33^ ^]^ Very recently, Ma's group innovatively applied this cutting‐edge technology to construct macrophage–tumor chimeric HMNVs.^[^ ^17^ ^]^ They first isolated the nuclei from four types of tumor cells (E.G7 mouse lymphoma tumor cells, 4T1 mouse triple‐negative breast cancer cells, B16 mouse melanoma cells, and MDA‐MB‐231 human triple‐negative breast cancer cells) of clinical patients, then introduced the nuclei into the nucleus‐removing M1‐type macrophages respectively, and finally extracted the chimeric HMNVs from the purified chimeras. Notably, macrophage–tumor hybrid cells could be first produced by transplanting tumor cell nucleus into activated M1‐type macrophages. Afterward, the hybrid chimeric cells will yield chimeric nanovesicles (aMT‐exos) just as normal cells naturally secrete EVs. As expected, the macrophage–tumor chimeric aMT‐exos showed remarkable immunotherapy outcomes for both primary and metastatic tumors, which provided an idea for the exploitation of new biotechnologies for HMNVs preparation.^[^ ^34^ ^]^

In short, the strategies for constructing different HMNVs are being expanded and updated. The integration of multiple preparation methods has not only improved the yield of hybrid nanovesicles but also endowed them with diverse functions, which have greatly promoted the practical applications of HMNVs in biology and biomedicine.

## 3 Classification of the HMNVs Based on Membrane Sources

**Figure 4:**
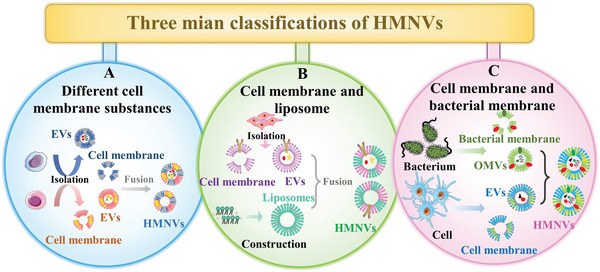
Schematic diagram of HMNVs classification based on the different sources of membrane components, which includes A) HMNVs made by a fusion of homologous or heterologous cell membrane substances, B) HMNVs made by a fusion of cell membrane and liposome, and C) HMNVs made by a fusion of cell membrane and bacterial membrane.

HMNVs have extended new horizons for applications of natural EVs in drug delivery and clinical treatments. Compared to the naturally secreted simplex EVs, the HMNVs become more stable in the physiological environment while showing extended circulation time, enhanced antiphagocytosis and specific targeting abilities, and more possibilities for various biological applications. With the rapid advancement of HMNVs fabrication technologies, increasing types of HMNVs have been developed. Herein, according to the different sources of membrane components, we divide the HMNVs into three major categories, which include 1) HMNVs made by a fusion of homologous or heterologous membrane substances, 2) HMNVs made by a fusion of cell membrane and liposome, as well as 3) HMNVs made by chimera biotechnology (**Figure** **4**).

### 3.1 HMNVs Fused by Homologous or Heterologous Cell Membrane Components

Currently, HMNVs are mainly produced by different types of cell membranes which can inherit diverse biological functions from homologous or heterologous source cells. For example, it is well‐documented that erythrocytes can be widely applied as promising drug delivery vehicles because of their inherent biocompatibility.^[^ ^13^ ^]^ Notably, damaged or senescent erythrocytes could promote the uptake of antigen‐presenting cells (APCs) and accordingly enhance the antitumor outcomes of macrophages and DCs.^[^ ^35^ ^]^ Therefore, Han et al. fused erythrocyte membranes with antigens‐loaded tumor cell membranes (4TI cells or B16F10‐Luc cells) to form HMNVs to deliver tumor antigens into splenic APCs, thus eventually enhancing cancer immunotherapy.^[^ ^25^ ^]^ Very recently, Lin et al. constructed a biomimetic nanocomplex for overcoming clinical limitations of antirheumatic drug sinomenine hydrochloride for rheumatoid arthritis (RA) treatment, such as short half‐life, poor water‐dispersibility, and dose‐dependent side effects.^[^ ^36^ ^]^ Through a typical coextrusion procedure, erythrocyte membranes and macrophage membranes were successfully formed hybrid nanovesicles, which were further loaded with Prussian blue‐coated sinomenine nanoparticles. The as‐designed multifunctional nanocomplexes ultimately exhibited excellent drug accumulation ability at the arthritic sites and remarkable disease‐remitting outcomes on RA.

Apart from HMNVs constructed by cell membranes, numerous recent works have reported that HMNVs could also be formed by fusing different types of cell membrane‐derived nanovesicles. Although the HMNVs preparation way of membrane‐derived nanovesicles is much more complex when compared with the pure cell membrane‐extracted method, the HMVNs fused by cell membrane‐derived nanovesicles might exhibit more diversified biological functions because such HMVNs contain a variety of bioactive substances such as nucleic acid (DNA or RNA), proteins, lipids, and some soluble metabolites, which inherited from the parent nanovesicles during their biogenesis. For example, Meng et al. developed HMNVs for reinforcing antitumor immunity, where the hybrid nanovesicles were constructed by a fusion of two types of cellular vesicles which were derived from genetically engineered 4T1 and B16F10 cancer cells.^[^ ^37^ ^]^ The resultant HMNVs expressed both high‐affinity SIRPa variants and PD‐1 that were inherited from their source cancer cells, making them suitable for dually blocking the CD47/SIRPα and PD‐1/PD‐L1 pathways. Furthermore, Chen's group reported a hybrid nanovesicle integrated three different cell membrane vesicles for greatly preventing both tumor recurrence and metastasis.^[^ ^16^ ^]^ They first fabricated three types of nanovesicles from tumor cell membranes, M1 macrophage membranes, and PMVs. Then the three as‐obtained nanovesicles were mixed to form HMNVs, which could not only block the CD47/SIRPα signaling pathway but also promote M2‐to‐M1 reprogramming within tumor tissues, eventually leading to a remarkable effect of tumor immunotherapy.

In addition to what has been discussed above, some groups also fused the cell membrane with the naturally secreted EVs (or EVs’ membrane) to endow the hybrid nanovesicles with improved biocompatibility.^[^ ^38^ ^]^ For instance, Zhang et al. combined MSC‐Exos with monocyte membranes to build HMNVs that could target myocardial injury cells and promote tissue regeneration.^[^ ^39^ ^]^ In another case, Li et al. hybridized platelet cell membranes with MSC‐Exos to promote the polarization of M1‐type macrophages into anti‐inflammatory M2‐type cells, which significantly repaired myocardial injury.^[^ ^2b^ ^]^ Moreover, Cheng's team hybridized MSC‐Exos with PM to enhance injured heart targeting ability and promote angiogenesis while reducing inflammation, which provided a new therapeutic idea for the treatment of myocardial ischemia‐reperfusion injury.^[^ ^32^ ^]^ Recently, this team further uncovered that membrane‐wrapped exosomes could also be utilized as a promising therapeutic hybrid platform.^[^ ^21^ ^]^ They prepared the HMNVs with cardiac targeting capability by encapsulating MSC‐Exos with platelet cell membranes for the treatment of myocardial infarction injury, which provides a powerful biomimetic modification strategy instead of chemical conjugating specific markers on the membrane of EVs.

### 3.2 HMNVs Fused by Cell Membranes and Liposomes

Although cell membranes‐derived hybrid nanovesicles are widely used in various biomedical applications, the limitations including low drug loading capacity, unsatisfactory stability, and complicated preparation processes still hinder their future development. Liposomes, a kind of small artificial vesicle fabricated by some standard methods such as thin‐film hydration, ultrasonic dispersion, and microfluidic systems, have a stable lipid bilayer structure similar to EVs. Due to the mature preparation procedure, excellent drug‐carrying property, low systemic toxicity, and adaptable features, liposomes have been frequently used as building blocks to construct HMNVs.^[^ ^40^ ^]^ On the one hand, liposomes can be directly mixed with different cell membranes to form hybrid nanovesicles. For example, Chen et al. produced anti‐inflammatory HMNVs by fusing the PEG‐modified liposomes with neutrophil membranes with the nature of inflammatory chemotaxis to target the acute myocardial injury sites and eliminate inflammation.^[^ ^41^ ^]^ Besides, Deng et al. fused the Celastrol‐loaded PEGylated liposomes with the DC2.4 cell membranes to greatly enhance Celastrol tumor accumulation and efficiently treat KRAS‐mutated pancreatic cancer both in vitro and in vivo.^[^ ^42^ ^]^

On the other hand, outside of the incorporation strategy between liposomes and various cell membranes, the construction of HMNVs by fusing liposomes with the preformed EVs is also a hot research topic in the hybrid vesicle field. For instance, Han et al. reported HMNVs hybridized by the photothermal sensitive liposomes and the M1‐Exos to cocarry Granzyme B (GrB) and SerpinB9 (Sb9) siRNA, in which the GrB was a key killer during immunotherapy while the Sb9 siRNA could enhance the GrB sensitivity to tumor cells by silencing Sb9, thus achieving significant combinational tumor therapy.^[^ ^43^ ^]^ Rayamajhi et al. fused the synthetic liposomes with the macrophages‐derived EVs to obtain tumor‐targeting HMNVs, which were further loaded with clinical drug doxorubicin to efficiently treat breast cancer with reduced side effects.^[^ ^22^ ^]^ In addition to small drug molecules, Lin et al. successfully loaded large nucleic acids such as the CRISPR/Cas9 system into HMNVs, which were generated by simply incubating the HEK293FT‐Exos with liposomes.^[^ ^26^ ^]^ As expected, the resultant HMNVs encapsulated with CRISPR/Cas9 plasmids could be successfully endocytosed by MSCs and manipulate the target gene expression in MSCs.

### 3.3 Cell Membranes and Bacterial Membranes

**Figure 5:**
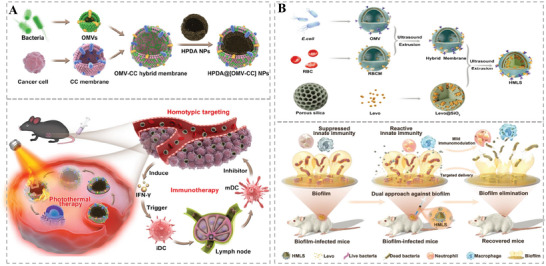
A) Schematic illustration of the bacterial outer membrane‐based HMNVs in preparation and antitumor effect. Reproduced with permission.^[^ ^45^ ^]^ Copyright 2020, American Chemical Society. B) Schematic illustration of the bacterial outer membrane‐based HMNVs in preparation and anti‐infection effect. Reproduced with permission.^[^ ^47^ ^]^ Copyright 2023, Wiley‐VCH.

In addition to the above‐mentioned fusion between eukaryotic cell membranes, prokaryotic bacterial membranes especially bacterial outer membrane vesicles (OMVs) are also significant components for the preparation of HMNVs. OMVs, whose biological characteristics are highly similar to the bacterial outer membrane, are one of the main ways of bacterial survival defense. Existing studies have reported that the OMVs contain a variety of biological activities, originating from outer membrane proteins, lipopolysaccharides, adhesins, immunomodulatory compounds, etc., which play a significant role in signal transduction, immune stimulation, and homologous targeting.^[^ ^44^ ^]^ Studies have found that OMVs tend to accumulate at the site of infection and have excellent immunostimulatory features. Based on this, Zhang and co‐workers fused *Escherichia coli* DH5α OMVs with B16‐F10 tumor cell membrane, and then coated on photothermal agent polydopamine nanoparticles, which could be applied to tumor photothermal treatment.^[^ ^45^ ^]^ As shown in **Figure** **5**A, the resultant HMNVs could specifically target the melanoma region, trigger tumor immune response, and subsequently cooperate with photothermal therapy to play a long‐term and significant tumor destruction. Similarly, Tang's team collaboratively constructed a tumor‐immunogenic and self‐adjuvanting HMNVs by fusing attenuated *Salmonella* OMVs and B16‐F10 melanoma cell membrane vesicles, which enhanced the flexible expandability and effectiveness of anticancer vaccination.^[^ ^46^ ^]^ Very recently, Zhuang et al. combined *E. coli* OMVs with plant‐derived thylakoid membranes (Tk) to prepare HMNVs with the tumor inflammation targeting for photodynamic effects‐boosted immunotherapy. ^[^ ^18^ ^]^

To expand the biomedical application of HMNVs based on bacterial membrane preparation, Chen et al. generated HMNVs of *E. coli* OMVs and erythrocyte membrane, and coated on silica nanoparticles containing antibiotics to realize the antibacterial treatment of transplant infection.^[^ ^47^ ^]^ Figure 5B shows the preparation and synergistic therapeutic mechanism of such HMNVs. Attractively, the OMV could not only endow the HMNVs with homotypic targeting effect but also induce a potent immunogenicity to achieve the antibacterial immune responses, while the erythrocyte membrane could reduce the immunogenicity from the OMVs, thus preventing extreme inflammatory stimulation. The synergistic strategy of hybrid membranes provides a promising option for the treatment of intractable bacterial inflammatory diseases such as implant infections.

## 4 Biomedical Applications of HMNVs

### 4.1 HMNVs for Rapid Diagnosis

**Figure 6:**
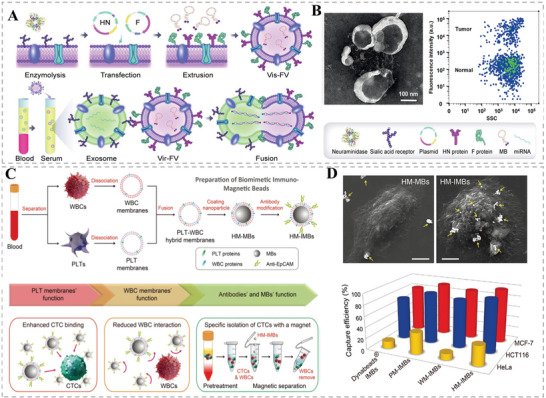
A) Schematic illustration of the fusion of exosome and Vir‐FVs for the rapid detection of exosomal miRNAs. B) TEM image of the fusion and the representative bivariate dot‐plots of Vir‐FVs after incubating with the serum of cancer patients and healthy donors. Reproduced with permission.^[^ ^15^ ^]^ Copyright 2019, Wiley‐VCH. C) Schematic illustration of the HMNVs‐coated nanoparticles (HM‐IMBs) for the rapid detection of circulating tumor cells (CTCs). D) SEM images of single MCF‐7 cell captured by HM‐MBs or HM‐IMBs and capture efficiency of various IMBs based on three types of cancer cells in cell culture medium. Reproduced with permission.^[^ ^52^ ^]^ Copyright 2018, Wiley‐VCH.

The naturally derived EVs have been widely used in the serological, imaging, and pathological diagnosis of different diseases owing to the abundant bioactive substances they contain and modifiable characteristics.^[^ ^48^ ^]^ As reported that high expression of arginase 1 protein (Arg1) in serum exosomes could lead to endothelial dysfunction in diabetic mice, so identifying the amount of Arg1 protein in serum EVs could be applied to monitor vascular homeostasis in diabetes.^[^ ^49^ ^]^ Besides proteins, rich miRNAs in EVs can regulate the expression of target genes, playing an indispensable role in the physiology and pathology of the organism, which could also be regarded as an important target of EVs for rapid disease diagnosis.^[^ ^50^ ^]^ For example, the circulating exosomal miRNA‐223 is one of the diagnostic biomarkers for acute ischemic stroke and the expression levels of miRNA‐1 and miRNA‐133a in serum exosomes are used to determine the degree of myocardial damage in cardiovascular disease patients.^[^ ^51^ ^]^ On these grounds, some elaborately designed HMNVs have emerged as rapid diagnosis and real‐time monitoring platforms for various diseases. As shown in **Figure** **6**A, Gao et al. successfully designed a virus‐mimicking fusogenic vesicle (Vir‐FV) for rapid detection of miRNAs in tumor exosomes employing genetic engineering.^[^ ^15^ ^]^ The researchers first constructed a cell membrane in which HN and F proteins were coexpressed and then loaded with molecular markers of exosomal miRNAs to obtain Vir‐FV. Finally, Vir‐FV was bound explicitly to the exosomes’ sialic acids and inserted into the target exosome membrane to form a detection fusion. After the signal markers bound to unique target miRNAs, efficient detection was completed within 2 h. This method is a highly promising diagnostic strategy that can complete the initial diagnosis of disease by comparing normal exosomes with tumor exosomes (Figure 6B). Moreover, HMNVs could also be used to identify circulating tumor cells (CTCs) in blood samples, which are crucial to early diagnosis of tumors.^[^ ^52^ ^]^ As presented in Figure 6C, the leukocyte membrane and the platelet membrane were fused to encapsulate magnetic nanoparticles. Afterward, the tumor cell‐related specific antibodies were conjugated on the surface of the hybrid membrane. The resultant hybrid vesicle‐camouflaged immunomagnetic beads (HM‐IMBs) could specifically capture CTCs and further isolate the pure CTCs in clinical blood samples with higher efficiency when compared with other separation methods (Figure 6D).

### 4.2 HMNVs for Precise Bioimaging

**Figure 7:**
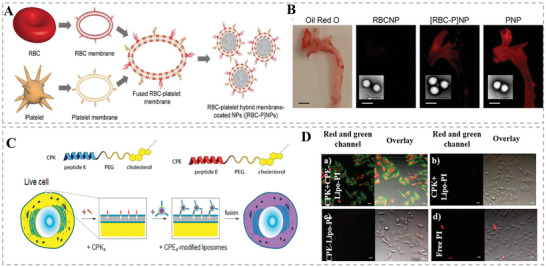
A,B) Schematic illustration of theragnostic TEVs for miRNA delivery and multimodel imaging. Reproduced with permission.^[^ ^13^ ^]^ Copyright 2017, Wiley‐VCH. C,D) Schematic diagram of the process of constructing and delivering PI dye by a pair of complementary coiled‐coil lipopeptides CPE4 and CPK4. Reproduced with permission.^[^ ^55^ ^]^ Copyright 2016, The Authors, published by American Chemical Society.

In recent decades, a variety of synthetic nanomaterials have been intensively exploited for bioimaging, showing high precision and favorable signal‐to‐noise ratio.^[^ ^53^ ^]^ It is worth mentioning that the superior cellular communication ability and natural or artificial vesicle structure of EVs and HMNVs provide perfect carriers for fluorescent dyes, imaging tracers, and nanoprobes, allowing these nanovesicles to be well applied in precise imaging.^[^ ^54^ ^]^ As displayed in **Figure** **7**A,B, Zhang et al. fused red blood cell (RBC) membrane with PMV to obtain custom‐tailored biomimetic HMNVs with favorable biological characteristics from their parent cells, which then coated on poly(lactic‐*co*‐glycolic acid) (PLGA) nanoparticles. ^[^ ^13^ ^]^ The fusion vesicles have remarkable biocompatibility and long in vivo circulation time. In particular, animal experiments have demonstrated that dye‐labeled HMNVs could selectively accumulate in atherosclerotic plaque sites and confirm the presence of atherosclerotic plaque within aortas by fluorescent imaging. To achieve efficient delivery of fluorescent dyes into living cells, Kros's group developed a new approach for the direct delivery of various molecules into the cytosol of live cells based on hybrid membrane fusion between lipopeptide CPE4‐decorated liposomes and lipopeptide CPK4‐modified live cells (Figure 7C,D).^[^ ^55^ ^]^ Notably, the CPE4 on liposomes and CPK4 on cell membranes are a pair of complementary coiled‐coil lipopeptides that could not only trigger the aforesaid targeted membrane fusion of liposomes with the live cell membranes but also concurrently liberate the encapsulated fluorescent dyes in liposomes, potentially for in vitro, ex vivo, and in vivo bioimaging applications.

### 4.3 HMNVs for Efficient Therapy of Various Diseases

As mentioned before, HMNVs combined the intrinsic advantages of natural vesicles and additionally acquired functions such as targeting ability and ideal drug loading capacity during artificial construction, making them being considered as powerful tools for highly efficient disease treatment.^[^ ^56^ ^]^ Below, we will briefly discuss the biomedical applications of HMNVs in the management of various diseases including cancer, cardiovascular diseases, inflammatory diseases, bacterial infections, and eye diseases.

#### 4.3.1 Cancer Treatment

**Figure 8:**
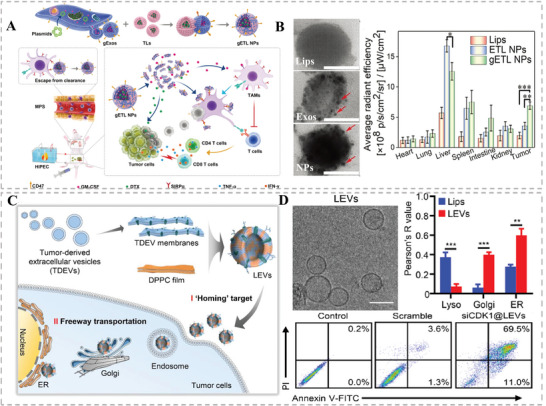
A,B) Schematic illustration and the excellent tumor targeting ability of the gETL NPs. Reproduced with permission.^[^ ^59^ ^]^ Copyright 2020, Wiley‐VCH. C,D) Schematic diagram of the preparation of stable hybrid lipid nanovesicles (LEVs) by fusing tumor‐derived EV (TDEV) membranes with phospholipids (DPPC) film to efficiently deliver siRNA to tumor sites with outstanding lysosome escape ability for boosting anti‐tumor therapy. Reproduced with permission.^[^ ^60^ ^]^ Copyright 2022, Wiley‐VCH.

Cancer is one of the severe diseases that endanger human health globally. Currently, radiotherapy, chemotherapy, and surgery are three major traditional treatment methods against cancer available in clinical practice. Although effective, these traditional clinical approaches have been greatly hindered by the problems such as poor targeting ability, serious side effects, high drug resistance, and potential tumor metastasis.^[^ ^57^ ^]^ Therefore, it is an urgent need to improve the traditional treatment methods while developing new therapeutic strategies to treat cancers. In this regard, HMNVs have been extensively implemented in cancer treatment by improving traditional chemotherapy or taking advantage of other new anticancer modalities.^[^ ^58^ ^]^ For example, to overcome the drug delivery hurdle and rapid drug resistance of hyperthermic intraperitoneal chemotherapy (HIPEC) toward metastatic peritoneal cancer (mPC), Lv et al. developed hybrid nanovesicles (gETL NPs) that fused by thermosensitive liposome and genetically engineered exosome (**Figure** **8**A,B).^[^ ^59^ ^]^ The gETL NPs were subsequently loaded with both docetaxel and granulocyte–macrophage colonies‐stimulating factor (GM‐CSF) for cooperating with the HIPE treatment. Such a combination strategy prominently increased drug penetration, promoted the repolarization of macrophages to M1, and finally inhibited tumor growth in CT26‐derived mPC xenografts. Very recently, Zhou et al. synthesized stable hybrid lipid nanovesicles (LEVs) by fusing tumor‐derived EV membranes with phospholipids (DPPC) film to efficiently deliver siRNA to tumor sites for boosting gene therapy (Figure 8C,D).^[^ ^60^ ^]^ Most importantly, compared to common lipid‐based carriers, the LEVs can precisely transport siRNA into tumor cells via the Golgi‐ and endoplasmic reticulum‐dependent pathways rather than the typical intracellular endosomal degradation transportation when delivering nucleic acid, thus promoting the tumor accumulation and efficient transfection of siRNA, as well as greatly enhancing the anticancer efficacy of LEVs in a hepatocellular carcinoma xenograft model.

In addition to chemotherapy and gene therapy, HMNVs have also frequently been utilized in photothermal therapy (PTT), immunotherapy, and combination therapy for cancer treatment.^[^ ^61^ ^]^ For instance, Jiang et al. incorporated a PTT agent Melanin into the hybrid nanovesicles fused by MCF‐7 cell membranes and erythrocyte membranes to simultaneously enhance the tumor‐targeting ability and blood circulation lifetime of the photothermal converters.^[^ ^62^ ^]^ Bu et al. encapsulated Fe_3_O_4_ into platelet‐cancer stem cell HMNVs to improve tumor targeting while enhancing the effect of PTT treatment of head and neck squamous cell carcinoma.^[^ ^58c^ ^]^ To integrate the PTT with chemotherapy, Wang et al. first constructed membrane nanovesicles derived from RBCs and B16‐F10 tumor cells (RBC‐B16 hybrid membranes), as well as chemotherapeutic drug doxorubicin (DOX)‐loaded PTT agent CuS nanoparticles (DCuS), respectively.^[^ ^14^ ^]^ Then, the DCuS were further camouflaged by the RBC‐B16 hybrid membranes to constitute DCuS@[RBC‐B16] NPs. As such, in comparison with the uncoated CuS NPs, the biomimetic CuS NPs exhibited longer circulation lifetime and improved homotypic‐targeting capabilities inherited from the source cells, resulting in remarkable chemo‐photothermal therapy of melanoma.

**Figure 9:**
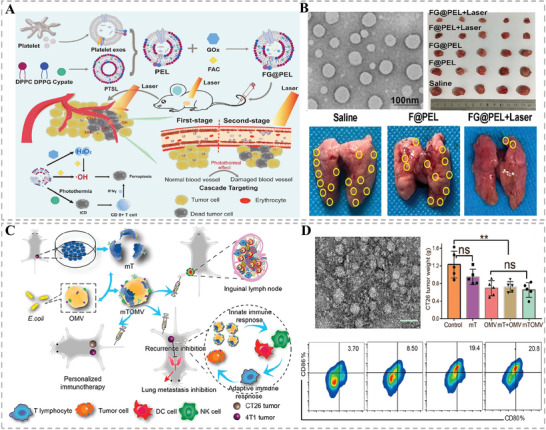
A) Schematic illustration of the preparation of HMNVs (FG@PEL) for a cascade synergetic anticancer therapy. B) TEM image of the FG@PEL and their great antitumor effect under laser irradiation. Reproduced with permission.^[^ ^31^ ^]^ Copyright 2022, Elsevier. C) Schematic diagram of the preparation of mTOMV and their multifaceted antitumor effects. D) TEM image and the effects of regulating the immune microenvironment. Reproduced with permission.^[^ ^64^ ^]^ Copyright 2021, American Chemical Society.

On the other hand, immunotherapy currently under clinical and preclinical investigation has revolutionized cancer therapy because it is possible to achieve cancer eradication treatment by activating antitumor immune cell populations in tumor sites and remodeling the tumor immunosuppressive microenvironment.^[^ ^63^ ^]^ First of all, immunotherapy could be induced by other treatment modalities such as PTT. Very recently, Ma et al. first hybridized platelet exos with photothermal sensitive liposomes and then embedded ferric ammonium (FAC) and glucose oxidase (GOx) to construct a dually targeted nanoplatform (FG@PEL) for a cascade synergetic anticancer therapy (**Figure** **9**A,B).^[^ ^31^ ^]^ More intriguingly, the PTT outcome triggered by Cypate, a photothermal agent incorporated into bilayer of liposomes, could induce in vivo immune response with an upregulated tumor infiltrating T cell and increased secretion of INFγ, which would augment the sensitivity of ferroptosis caused by GOx and FAC, eventually achieving a remarkably synergistic effect for tumor therapy. Furthermore, to directly activate the immune system for personalized immunotherapy, various membrane modules including autologous tumor cell membrane and OMVs secreted by Gram‐negative bacteria could be used as tumor antigens or adjuvants to enhance the immunogenicity. Zhang's group fused the tumor cell membrane (mT) with the bacterial OMV to constitute new multifunctional nanovesicles mTOMV for concurrently enhancing both innate and adaptive immune responses in bilateral tumor model (Figure 9C,D).^[^ ^64^ ^]^ Encouragingly, the resultant mTOMV with great biocompatibility not only suppressed primary tumor growth but also inhibited tumor lung metastasis, providing great potential for clinical translations.

#### 4.3.2 Cardiovascular Diseases Treatment

**Figure 10:**
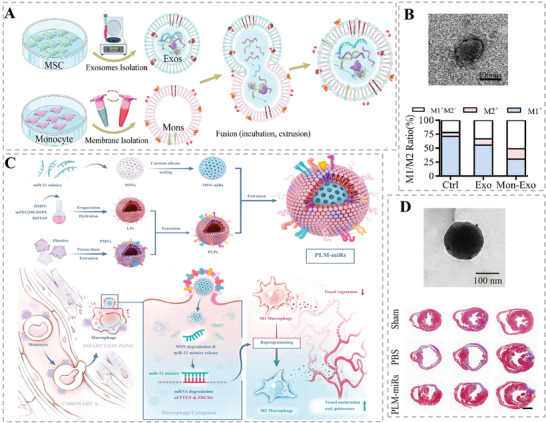
A) Schematic illustration of the synthesis process and B) the TEM image as well as the therapeutic effect of the Mon‐Exos for myocardial injury. Reproduced with permission.^[^ ^39^ ^]^ Copyright 2020, Elsevier. C) Schematic illustration of the construction of fused nanovesicles (PLM‐miRs) and D) their TEM image as well as anti‐inflammatory effects in myocardial injury. Reproduced with permission.^[^ ^67^ ^]^ Copyright 2021, Wiley‐VCH.

Cardiovascular disease, a class of disorders of the heart or blood vessels, is one of the leading causes of death and disability worldwide, often accompanied by symptoms of myocardial infarction (MI), heart failure, or strokes. At present, surgical treatment and drug intervention are mainly clinical strategies against cardiovascular disease, among which surgical treatment is risky and expensive while drug therapy shows low bioavailability and poor lesion‐targeting ability. Hence, these shortcomings become critical barriers to the treatment of cardiovascular disease.^[^ ^65^ ^]^ Fortunately, mounting studies have proved that naturally derived EVs could promote vascular regeneration, eliminate inflammation, and protect blood vessels, which indicate that the artificially fabricated HMNVs inheriting EVs biological features also have excellent prospects for the treatment of cardiovascular disease.^[^ ^41^, ^66^ ^]^ For example, Zhang et al. designed a pro‐regenerative hybrid nanovesicle (Mon‐Exos), which fused the MSC EVs with the monocyte–macrophage membrane to promote cardiac repair and cardiac functional remodeling (**Figure** **10**A,B).^[^ ^39^ ^]^ Notably, the embedded monocyte membrane components endowed the Mon‐Exos with high specific targeting capability to injured myocardium both in vitro and in vivo due to the recruitment nature of monocytes after heart injury. Similarly, to reduce immune phagocytosis and repair cardiac injury, Li et al. decorated the PMV to MSC EVs via the fusion–extrusion method to form a hybrid nanovesicle, which gratifyingly integrating the injured endothelium targeting ability of the platelets and proangiogenic functions of the MSC EVs.^[^ ^21^ ^]^

In recent years, more and more researchers have found that hybrid nanovesicles could promote the M1–M2 repolarization of macrophage, alleviate inflammation, and play a multifaceted role in cardiac repair which are attributed to the multiple internal miRNAs and bioactive substances inheriting from the source cells.^[^ ^2^, ^39^, ^67^ ^]^ Tan et al. prepared platelet‐like HMNVs that fused platelet‐derived vesicles with liposomes while encapsulating the mesoporous silica spheres loaded with the anti‐inflammatory miRNA‐21 for targeted treatment of MI‐reperfusion injury (Figure 10C,D).^[^ ^67^ ^]^ Attractively, the as‐fabricated fusions could target monocytes or macrophages in the blood circulation and converge on myocardial vascular lesions to perform multiple therapeutic effects including reprogramming the M1‐type macrophages into M2‐type, releasing anti‐inflammatory factors to repair vascular damage, and ultimately preserving the cardiac function of MI mice. With the increasing applications of HMNVs with naturally favorable properties and acquired versatile properties in cardiovascular therapeutic research, the avenues for targeted drug utilization, cardiac repair, and cardiovascular microenvironment regulation have been broadened.

#### 4.3.3 Inflammatory Diseases Treatment

The complex inflammatory process is a naturally protective or defensive response to a wide variety of imbalances such as trauma, infection, toxins, autoimmune injury, and other adverse reactions, leading to a recovery of tissue homeostasis. However, dysregulated and uncontrolled inflammation can become destructive to the host, which contributes to plenty of inflammatory diseases including rheumatoid arthritis (RA), hepatitis, inflammatory bowel disease, pancreatitis, and atherosclerosis. Therefore, anti‐inflammatory therapy has been regarded as a promising strategy to treat the aforementioned relevant diseases.^[^ ^68^ ^]^ As is well‐known, macrophages are essential regulatory cells in the development of inflammation, which generally include two types. One is a proinflammatory M1 phenotype that can secrete inflammatory factors and promote inflammation and the other one is an anti‐inflammatory M2 phenotype that can secrete cytokines such as IL‐10, IL‐rα, and TGF‐β for potential anti‐inflammation therapy.^[^ ^69^ ^]^ Most importantly, under certain stimulation, the two types can be converted into each other. Encouragingly, HMNVs carrying a variety of bioactive substances originating from the sources cells can regulate the secretion of inflammatory factors and correspondingly reprogram macrophage types from M1 to M2, which play an invaluable role in alleviating inflammation in various diseases.^[^ ^70^ ^]^

**Figure 11:**
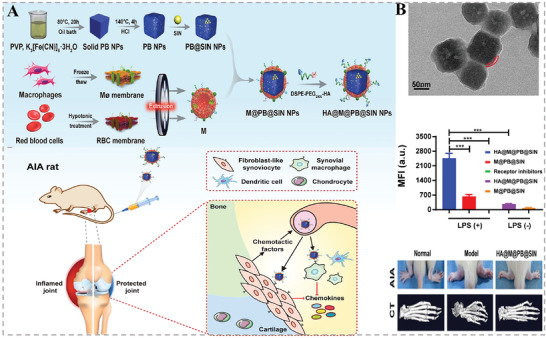
A) Schematic illustration of the preparation of HA@M@PB@SIN NPs. B) TEM image and a significant inhibitory effect on inflammation in the treatment of RA of the HA@M@PB@SIN NPs. Reproduced with permission.^[^ ^36^ ^]^ Copyright 2022, Elsevier.

RA is a typical autoimmune disease commonly attributed to persistent inflammation, which would cause irreversibly inflamed joints, such as cartilage degradation and bone erosion. Meanwhile, various immune cells such as macrophages, fibroblast‐like synoviocytes (FLSs), and neutrophils are rich in rheumatoid synovial joints. Very recently, to improve the therapeutic efficacy of an antirheumatic drug sinomenine hydrochloride (SIN), Lin et al. constructed multifunctional ROS‐scavenging Prussian blue nanoparticles (PB NPs) loaded with SIN for the treatment of RA by regulation of proinflammatory cytokines.^[^ ^36^ ^]^ Notably, the resultant SIN‐loaded PB NPs were further camouflaged by hybrid nanovesicles deriving from macrophage membranes and erythrocyte membranes (M@PB@SIN NPs), in which the macrophage membranes endowed the NPs with macrophages‐targeting capability while the erythrocyte membranes enabled their good biocompatibility and prolonged circulation time (**Figure** **11**A). Cooperated with the inserted DSPE‐PEG2000‐HA into the nanovesicles membrane, the biomimetic NPs could precisely target inflammatory FLSs and macrophages in the damaged joints, eventually significantly inhibiting the secretion of various proinflammatory factors with great biosafety to relieve RA symptoms in the rat model of adjuvant‐induced arthritis (Figure 11B). Overall, in the course of its therapeutic effects, the HMNVs could promote the repolarization of M1 cells into M2 cells and further shown a broad‐spectrum anti‐inflammatory effect, which provided a promising approach for arthritis treatment.

**Figure 12:**
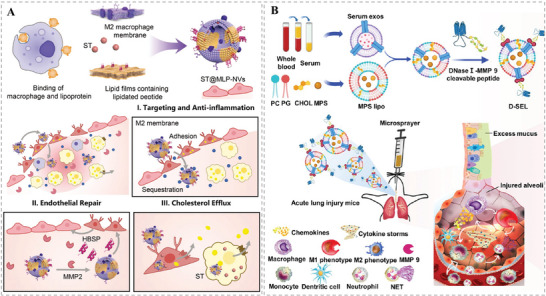
A) Schematic illustration of the preparation and anti‐inflammatory repair effect of MLP‐NVs. Reproduced with permission.^[^ ^73^ ^]^ Copyright 2022, Wiley‐VCH. B) Schematic illustration of the preparation and significant inhibitory effect on inflammation in the treatment of ALI or ARDS of the D‐SEL. Reproduced with permission.^[^ ^75^ ^]^ Copyright 2023, American Chemical Society.

Atherosclerosis, with high morbidity and mortality, is a major contributor to vascular disease. Various pathogenic factors such as vascular endothelial injury, inflammation, and lipid deposition drive the development of AS.^[^ ^71^ ^]^ Currently, traditional treatments, which focus on controlling coagulation and thrombosis as well as improving hypertension and hyperlipidemia, still have the challenges of limited drug delivery routes, rapid drug clearance, and poor bioavailability, which in turn affects the therapeutic efficacy of AS.^[^ ^72^ ^]^ To address these issues, Zhang and co‐workers fused M2‐type macrophage membranes with lipidated peptide (DOPE‐pp‐HBSP), simultaneously encapsulating the cholesterol‐lowering drug simvastatin (ST), and successfully prepared a biomimetic HMNVs (MLP‐NVs).^[^ ^73^ ^]^ As shown in **Figure** **12**A, the MLP‐NVs not only exerted the intrinsic inflammation‐targeting ability and anti‐inflammatory effects of M2‐type macrophage, but also mimicked lipoproteins to achieve a matrix metalloprotease 2 (MMP2)‐responsive release of the helical b‐surface peptides (HBSPs) to promote functional repair of endothelial cells. Afterward, the encapsulated ST would be liberated and play a role of promoting cholesterol efflux. Collectively, the multicomponents synergistically exerted a remarkable therapeutic effect on AS.

Severe inflammatory dysregulation and grievous lung tissue damage are present in acute lung injury (ALI) or acute respiratory distress syndrome (ARDS).^[^ ^74^ ^]^ To control excessive inflammation and repair lung function, Zheng et al. designed a smart inhalable nanoplatform (D‐SEL) that could break the synergistic action of neutrophils and macrophages through degrading dysregulated neutrophil extracellular traps (NETs) and repolarizing lung macrophages toward the M2 phenotype, thus eventually displaying promising combinatorial treatment of ALI (Figure 12B).^[^ ^75^ ^]^ Specifically, a hybrid nanocarrier (termed SEL) was first fused from serum exosomes and liposomes, which were loaded with the therapeutic drug methylprednisolone sodium succinate (MPS). Then, DNase I was conjugated on the surface of the as‐fabricated SEL via an MMP‐9‐cleavable peptide, resulting in the successful fabrication of the inhalable nanoplatform (MPS/D‐SEL), which could exhibit a synergetic anti‐inflammatory efficacy inducing NETs degradation, suppressing neutrophil activation, and promoting M2 macrophage polarization. As such, the MPS/D‐SEL with high drug loading capacity could play an important role in regulating the inflammatory environment and facilitating the repair of damaged lung tissues, showing great promise for the treatment of acute lung inflammation.

**Figure 13:**
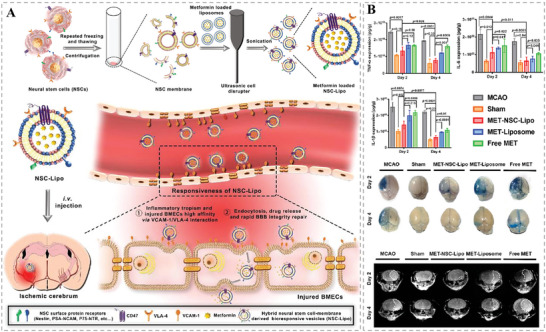
A) Schematic illustration of the preparation of NSC‐lipo HMNVs for inflamed BBB targeting and brain drug delivery. B) The effective anti‐inflammatory and BBB repair effects of MET‐NSC‐lipo. Reproduced with permission.^[^ ^77^ ^]^ Copyright 2023, Elsevier.

The blood‐brain barrier (BBB) is a unique barrier that maintains homeostasis of the brain's internal environment. However, the destruction of BBB integrity often results in infiltration of leukocytes within the brain and disruption of the microenvironment, leading to a number of acute and chronic inflammatory encephalopathies, such as ischemic stroke, Alzheimer's disease, and Parkinson's disease.^[^ ^76^ ^]^ Therefore, effective targeting of the inflamed BBB and timely repair of its integrity are essential for the treatment of inflammatory brain diseases. It has been found that brain microvessel endothelial cells (BMECs) can accelerate BBB injury. Based on this, Gao and co‐workers fused neural stem cell (NSC) membranes with liposomes while loaded with metformin to prepare hybrid bioresponsive vesicles (NSC‐lipo) that could target damaged BMECs and subsequently be smoothly endocytosed by inflammatory lesion areas.^[^ ^77^ ^]^ Results revealed that the HMNVs had a high drug loading capacity, which could achieve targeted aggregation at the inflammatory site, downregulate the inflammatory response of BMECs, promote the repair of BBB, and initiate the neuroprotective mechanism, ultimately improving the survival rate of mice with ischemic stroke disease (**Figure** **13**A,B). This study provides a pathway for brain‐targeted drug delivery and expands the application of HMNVs in the treatment of inflammatory brain diseases.

#### 4.3.4 Bacterial Infections Treatment

**Figure 14:**
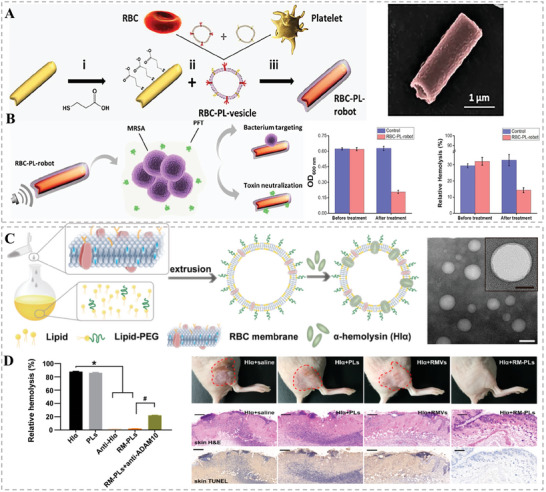
A) Schematic diagram of the synthesis process of RBC‐PL‐robot B) and its bacteria targeting as well as toxin neutralization abilities. Reproduced with permission.^[^ ^79^ ^]^ Copyright 2018, AAAS C) Schematic illustration of the preparation process and TEM morphology characterization of the HMNVs (RM‐PLs). D) The excellent ability of the resultant RM‐PLs to clear α‐hemolysin (Hlα) and repair in vivo Hlα‐induced damage. Reproduced with permission.^[^ ^80^ ^]^ Copyright 2019, American Chemical Society.

Bacterial infections are increasingly recognized as a considerable cause of morbidity and mortality, posing a big threat to public health all over the world. Pore‐forming toxins (PFTs), a class of hemolytic protein toxins that are normally produced by pathogens and released into the bloodstream, are major factors of bacterial pathogenesis causing cell lysis and life threat. Based on this notion, PFTs would be an ideal therapeutic target for the efficient treatment of bacterial infections.^[^ ^78^ ^]^ In 2018, Wang's groups worked together to develop ultrasound‐propelled hybrid membrane‐cloaked nanorobots for targeted neutralization of pathogenic toxins and specific elimination of pathogenic bacteria (**Figure** **14**A,B).^[^ ^79^ ^]^ Specifically, the biomimetic nanorobots were constructed with gold nanowires as the acoustic core and camouflaged with RBC‐PL vesicles derived from RBC membranes and platelet (PL) membranes. Such hybrid RBC–PL membranes inherited a wealth of functional proteins from both source cells which enabled the as‐fabricated nanorobots to synchronously absorb and clear PL‐bound pathogenic bacteria and RBC‐targeted PFTs secreted by the bacteria. To further improve the detoxification of HMNVs to bacterial toxins, He et al. hybridized artificial lipid membranes with RBC membranes to remove bacteria‐secreted PFTs.^[^ ^80^ ^]^ As presented in Figure 14C, with easy production procedures, the PEGylated lipids were fused in the RBC membranes to form the HMNVs (RM‐PLs) for strengthening the detoxification capacities. Notably, the incorporated lipids could greatly stabilize the hybrid membranes in a complex physiological environment. As such, the resultant RM‐PLs could effectively sequester a model bacterial toxin α‐hemolysin (Hlα) and significantly reduced the in vivo Hlα‐induced damage, providing a promising biomimetic nanoplatform of HMNVs for biomedical applications (Figure 14D).

#### 4.3.5 Eye Diseases Treatment

**Figure 15:**
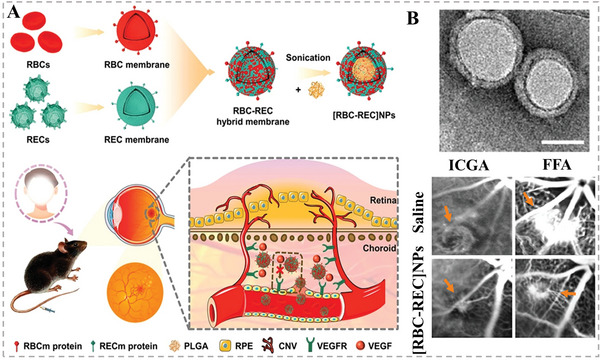
A) Schematic illustration of the preparation of hybrid biomimetic nanoparticles ([RBC‐REC] NPs) for noninvasive targeted treatment of laser‐induced CNV. B) TEM image of the [RBC‐REC] NPs and their representative fundus photographs (FFA and ICG) of CNV regions on day 14 after treatment. Reproduced with permission.^[^ ^81b^ ^]^ Copyright 2021, American Chemical Society.

In addition to the above disease types, HMNVs are widely available for therapeutic studies of other multiple diseases.^[^ ^81^ ^]^ For example, Li et al. developed hybrid nanovesicles‐based antiangiogenic nanoagents for noninvasively targeted treatment of choroidal neovascularization (CNV), which is a leading cause of blinding diseases and currently is managed by invasively injecting antiangiogenic agents into vitreous body.^[^ ^81b^ ^]^ As shown in **Figure** **15**A,B, the biomimetic antiangiogenic nanoagents were constructed by cloaking hybrid membranes on PLGA NPs, ultimately termed as [RBCREC]NPs, where the hybrid membranes were fused from erythrocyte membranes and retinal endotheliocyte membranes which respectively endowed the NPs with immune evasion as well as self‐recognition capability to target retinal endotheliocytes. More attractively, the vascular endothelial growth factor receptor (VEGFR) highly expressed on the retinal endotheliocyte membranes further provided the [RBCREC]NPs a competitive inhibition effect between the vascular endothelial growth factor (VEGF) and the VEGFR on host retinal endotheliocytes. Finally, the biomimetic anti‐VEGF NPs exhibited a considerable reduction of the leakage and proportion of CNV by a noninvasively intravenous treatment in a laser‐induced CNV mouse model. In short, HMNVs, with ideal properties integrated from natural cell sources and subsequent artificial addition, provide a safer and more efficient strategy for the treatment of various diseases. **Table** **2** summarized different biomedical applications of HMNVs, disease types, fusion targets, contents, and advantage.

**Table 2:** Different biomedical applications of the HMNVs.

Biomedical applications	Disease types		Fusion components	Exogenously loaded substances	Advantages	Refs.
Diagnosis	Cancer		Virus‐mimicking fusogenic vesicle–tumor exosome	Signal markers	Rapid detection of tumor miRNAs	[15]
	Cancer		Leukocyte membrane– platelet membrane	None	Specific capture of the CTCs	[52]
Bioimaging	Normal cells and tissues		RBC membrane– platelet membrane	PLGA	Remarkable biocompatibility, long circulation time	[13]
	Normal Zebrafish embryos		CPE4‐ modified liposome– CPK4‐ modified cell membrane	Fluorescent dyes	Excellent bioimaging ability in vitro, ex vivo, and in vivo	[55]
Disease treatment	Cancer	Metastatic peritoneal cancer	Thermosensitive liposome– engineered exosome	DTX, GM‐CSF	Cooperated with the HIPEC, enhanced drug penetration, and repolarizing effect	[59]
		Hepatocellular carcinoma	Tumor‐derived EV membrane– phospholipids film	siRNA	Efficient gene therapy, great tumor growth inhibition outcome	[60]
		Breast cancer	MCF‐7 cell membrane– erythrocyte membrane	Melanin	Tumor targeting ability, long circulation time, cooperated with PTT	[62]
		Head and neck squamous cell carcinoma	Patelet membrane–cancer stem cell membrane	Fe_3_O_4_	Enhanced tumor targeting ability, cooperated with PTT	[58c]
		Melanoma	RBC membrane–B16‐F10 tumor cell membrane	Doxorubicin, CuS nanoparticles	Long circulation time, homotypic‐targeting capability, remarkable chemo‐PTT	[14]
		Melanoma	Platelet exosome– photothermal sensitive liposome	Ferric ammonium, glucose oxidase	Activation of in vivo immune response, sensitive to ferroptosis, great tumor growth inhibition outcome	[31]
		Bilateral tumor	Tumor cell membrane–outer membrane vesicle	None	Enhaced innate and adaptive immune responses, inhibition of tumor growth and lung metastasis	[64]
	Cardiovascular diseases	Ischemic heart diseases/myocardial infarction	MSC EVs– monocyte–macrophage membrane	None	Promoted cardiac repair capacity, inflammation targeting capability	[39]
			Platelet membrane–MSC EVs	None	Cardiac injury repair capacity, endothelium targeting ability, angiogenesis	[21]
			Platelet‐derived vesicle‐ liposome	Mesoporous silica spheres, anti‐inflammatory miRNA‐21	Targeting ability to monocytes or macrophages, repolarizing effect, anti‐inflammatory factors release, ability to repair vascular damage	[67]
	Inflammatory diseases	Rheumatoid arthritis	Macrophage membrane– erythrocyte membrane	Prussian blue nanoparticles, hydrochloride	Target macrophages, inhibit inflammatory factors, long circulation, great biosafety	[36]
		Atherosclerosis	M2‐type macrophage membrane– lipidated peptide	Simvastatin	Target inflammation site, promote endothelial cells repair, promote cholesterol efflux, anti‐inflammatory	[73]
		Acute lung injury/acute respiratory distress syndrome	Serum exosome– liposome	Methylprednisolone sodium succinate	Enhanced delivery efficiency, regulate inflammatory environment, repair damaged lung tissues, repolarizing effect	[75]
		Ischemic stroke	Neural stem cell membrane– liposome	Metformin	Cross the BBB, target inflammatory, downregulate the inflammatory response, repair BBB	[77]
	Bacterial infections	Bacterial infections	RBC membrane– Platelet membrane	Gold nanowires	Neutralization of pathogenic toxins; Specific elimination of pathogenic bacteria	[79]
			PEGylated lipids–RBC membrane	None	Detoxification, sequester a model bacterial toxin Hlα, reduce the Hlα‐induced damage	[80]
	Eye diseases	Blinding diseases	Erythrocyte membrane–retinal endotheliocyte membrane	PLGA	Target CNV, reduce the leakage and area of CNV	[81b]

## 5 Ongoing Challenges and Future Perspectives of HMNVs

**Figure 16:**
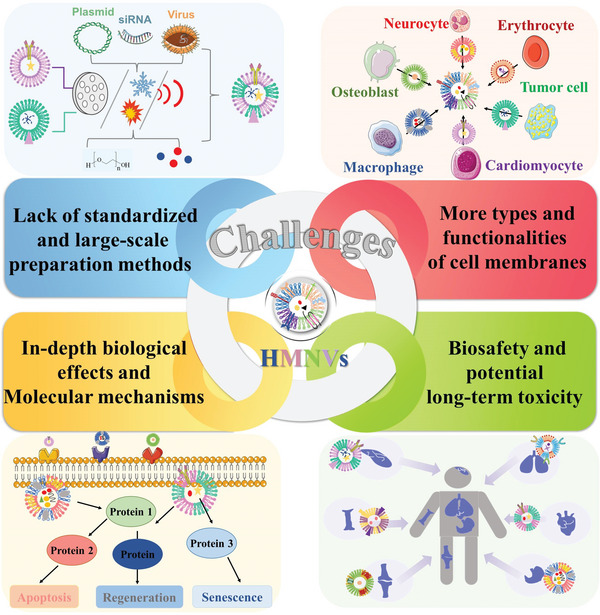
Current ongoing challenges and crucial limitations of the HMNVs for realizing their clinical practice.

As promising alternatives to EVs, hybrid membrane nanovesicles (HMNVs) have emerged as advanced platforms for the targeted delivery of different diagnostic agents and therapeutics.^[^ ^82^ ^]^ Strikingly, compared to the natural simplex EVs, the integration of natural source cells‐derived functions and artificial modification potentials can endow the HMNVs with precise targeting ability, sufficient production yield, high drug loading capacity, as well as possible additional multiple functions. However, such cutting‐edge hybrid membrane vesicles are still in their beginning stages and have not been applied in clinical practice. To expand the preclinical studies and realize the clinical practice of HMNVs, there are a number of essential challenges and crucial limitations that should be resolved, which are discussed in the following points (**Figure** **16**).

First, there is a lack of standardized and cost‐efficient large‐scale preparation methods and mature long‐term storage approaches to HMNVs. Although artificially assembled HMNVs have better production yield and controllability compared to naturally secreted EVs, scalability is still a critical issue need to be settled for their clinical utility. Specifically, in additional to obtaining sufficient membranes‐derived vesicles, the extraction techniques should also be optimized and carefully selected regarding the different applications of HMNVs, because the sophisticated preparation processes will affect the integrity and composition of cell membrane proteins. Therefore, researchers should compare diverse methods to summarize some standardized preparation steps to finally establish convenient, reliable, and repeatable manufacturing processes for the high‐throughput production of HMNVs.

Second, the potential of applying more types of cell membranes and their functionalities involved in HMNVs has not been fully exploited. Until now, hybrid membranes‐based vesicles have been widely fabricated by using various membranes of cancer cells, RBCs, mesenchymal stem cells, macrophages, platelets, endotheliocytes, and bacterial OMVs. Maybe membranes from other immune cells and plant cells with application prospects in biomedicine could also be developed as the building block for the construction of HMNVs. Furthermore, the cell type and membrane proportion should be elaborately considered to complement the functionalities of each component (e.g., cell membrane or liposome), further broadening the biomedical application of biomimetic HMNVs in the rapid diagnosis, precise bioimaging, and efficient therapy of various diseases.

Third, more in‐depth research on the biological effects as well as molecular mechanisms of HMNVs should be comprehensively investigated since the heterogeneous features of original source cells. Besides, incorporating artificial lipid membranes into HMNVs may cause unknown influences on the natural cell membranes, which would accordingly change their pristine biological functionalities. In this regard, more information on the biological characteristics of HMNVs as well as their detailed interactions with biomacromolecules from the cells or tissues of the body need to be further elucidated. Hence, apart from chemists and materials scientists, more biologists and basic medical scientists could devote mounting effort to addressing this fundamental issue of HMNVs.

Finally, in terms of the clinical translation of HMNVs, their biosafety and potential long‐term toxicity should be systematically evaluated. On the one hand, as previously mentioned, some biological features of HMNVs involved in their blood circulation, immune response, biodistribution, and elimination are still not completely explored. On the other hand, metabolic risks also exist in additionally integrated materials such as encapsulated nanoparticles, loaded nucleic acids, and other embedded biologically active substances. Thus, systematic assessment of the short‐term and long‐term biosafety as well as the fundamental principles of chemical–material–biological interactions on HMNVs need to be further thoroughly identified, which are essential for the rational design and clinical use of HMNVs in biomedicine.

## 6 Conclusions

In summary, this review focused on the current progress of the HMNVs for different biomedical applications. Particularly, diverse HMNVs preparation strategies, major types of HMNVs based on membrane composition, and their biological applications in rapid diagnosis, precise bioimaging, as well as efficient therapy of various diseases including cancer, cardiovascular diseases, inflammatory diseases, bacterial infections, and eye diseases were respectively outlined. In fact, the development of HMNVs is still in its preliminary stage and their clinical applications remained with several critical challenges. Encouragingly, HMNVs have tremendous commercial advantages and research potentials in biotechnology, nanomedicine, and healthcare, gradually filling the gap between preclinical research and clinical translation. We believe that the advanced HMNVs can serve as powerful but flexible biomimetic nanoplatforms for achieving personalized medicine and precision treatment.

## Conflict of Interest

The authors declare no conflict of interest.

## Author Contributions

M.S., M.H., K.S., and J.Z.gave the conceptualization. M.S. conducted the investigation and writing—original draft. J.Y., Y.F., Y.Z., and J.S. created the specific visualization. All the authors revised the manuscript. M.H., K.S., and J.Z. contributed to funding acquisition.
